# Pre‐Procedural Gamma‐Glutamyltransferase‐To‐Lymphocyte Ratio as an Adjunctive Biomarker for Differentiating Hunner‐Type Interstitial Cystitis From Bladder Pain Syndrome

**DOI:** 10.1111/iju.70638

**Published:** 2026-09-14

**Authors:** Shota Kakita, Tomohiro Matsuo, Shota Yamada, Asato Otsubo, Kyohei Araki, Kensuke Mitsunari, Kojiro Ohba, Hiroko Hayashi, Ryoichi Imamura

**Affiliations:** ^1^ Department of Urology Nagasaki University Graduate School of Biomedical Sciences Nagasaki Japan; ^2^ Department of Pathology Sasebo City General Hospital Sasebo Japan

**Keywords:** biomarker, bladder pain syndrome, gamma‐glutamyltransferase‐to‐lymphocyte ratio, Hunner lesion, interstitial cystitis

## Abstract

**Objectives:**

To evaluate the potential role of the pre‐procedural gamma‐glutamyltransferase‐to‐lymphocyte ratio (GLR) as an adjunctive biomarker for differentiating Hunner‐type interstitial cystitis (HIC) from bladder pain syndrome (BPS).

**Methods:**

Consecutive patients who underwent cystoscopic evaluation with hydrodistension for suspected interstitial cystitis/bladder pain syndrome between April 2016 and September 2025 were retrospectively analyzed. HIC and BPS were diagnosed by expert adjudication based on cystoscopic, clinical, and histopathological findings. GLR was calculated as serum gamma‐glutamyltransferase divided by peripheral lymphocyte count. Diagnostic performance was assessed using receiver operating characteristic analysis, and associations with HIC were evaluated using logistic regression.

**Results:**

Among 106 patients, 62 had HIC and 44 had BPS. GLR was significantly higher in HIC than BPS (21.4 ± 14.7 vs. 14.2 ± 15.2, *p* = 0.016) and correlated with total histological activity score (*ρ* = 0.606, *p* < 0.001). GLR showed an AUC of 0.759, while GGT showed similar discrimination (AUC 0.776; *p* = 0.592 vs. GLR). Adding GLR to a clinical model did not improve discrimination (AUC 0.948 vs. 0.949; *p* = 0.879). In the covariate‐adjusted multivariable analysis, GLR remained associated with HIC (odds ratio 1.04, 95% confidence interval 1.01–1.10, *p* = 0.032).

**Conclusions:**

Pre‐procedural GLR may serve as a practical blood‐based adjunct reflecting bladder‐inflammatory histopathology and supporting early phenotyping across the IC/BPS spectrum, although prospective external validation is required.

## 1 Introduction

Interstitial cystitis/bladder pain syndrome (IC/BPS) is a chronic condition characterized by bladder‐related pain, pressure, or discomfort accompanied by lower urinary tract symptoms in the absence of confusable diseases. Contemporary guidelines emphasize Hunner lesion status in IC/BPS classification, and cystoscopy remains the standard clinical method for identifying Hunner lesions [1]. In line with the Japanese guideline‐based disease concept, patients with IC/BPS and Hunner lesions were classified as having interstitial cystitis (IC), also referred to as Hunner‐type IC (HIC), whereas those without Hunner lesions were classified as having bladder pain syndrome (BPS) in the present study.

HIC is increasingly recognized as a distinct bladder‐centric inflammatory disease that differs from BPS in clinicopathological features and treatment response [1, 2]. Patients with Hunner lesions tend to be older and to have more severe bladder‐centric manifestations, including higher urinary frequency and reduced bladder capacity [2]. Accordingly, Hunner lesion status is important for diagnostic classification and therapeutic decision‐making [1, 3]. However, cystoscopy is invasive and resource‐dependent and may not be readily performed early in all patients with suspected IC/BPS.

Accumulating evidence supports a multifactorial IC/BPS model involving urothelial barrier dysfunction, immune activation, chronic inflammation, neural sensitization, and oxidative stress [4, 5, 6]. These mechanisms are particularly relevant to HIC, which is generally associated with more prominent bladder‐centric inflammation and epithelial injury than BPS [6, 7]. Therefore, biomarkers reflecting redox–immune balance may help identify patients likely to harbor Hunner lesions before invasive evaluation. Recent serum‐ and urine‐based biomarker studies further support the feasibility of phenotype‐related biomarker development in IC/BPS [8].

Gamma‐glutamyltransferase (GGT) is a routinely available laboratory parameter involved in glutathione homeostasis and oxidative stress biology [9, 10]. Peripheral lymphocyte count reflects host immune and inflammatory status, and the GGT‐to‐lymphocyte ratio (GLR) has been evaluated as a simple marker in several inflammation‐ and oxidative stress‐related conditions [11, 12]. However, whether GLR can serve as an adjunctive biomarker for differentiating HIC from BPS has not been established. This study aimed to evaluate pre‐procedural GLR as an accessible blood‐based adjunct for differentiating HIC from BPS and supporting early clinical phenotyping.

## 2 Materials and Methods

### 2.1 Study Design and Patients

This was a retrospective observational study of patients who underwent cystoscopic evaluation with hydrodistension for suspected IC/BPS at Nagasaki University Hospital. The study protocol was approved by the institutional ethics committee (approval No. 20122138) and conducted in accordance with the Declaration of Helsinki. Given the retrospective design, written informed consent was waived, and an opt‐out option was provided via the institutional website.

**FIGURE 1:**
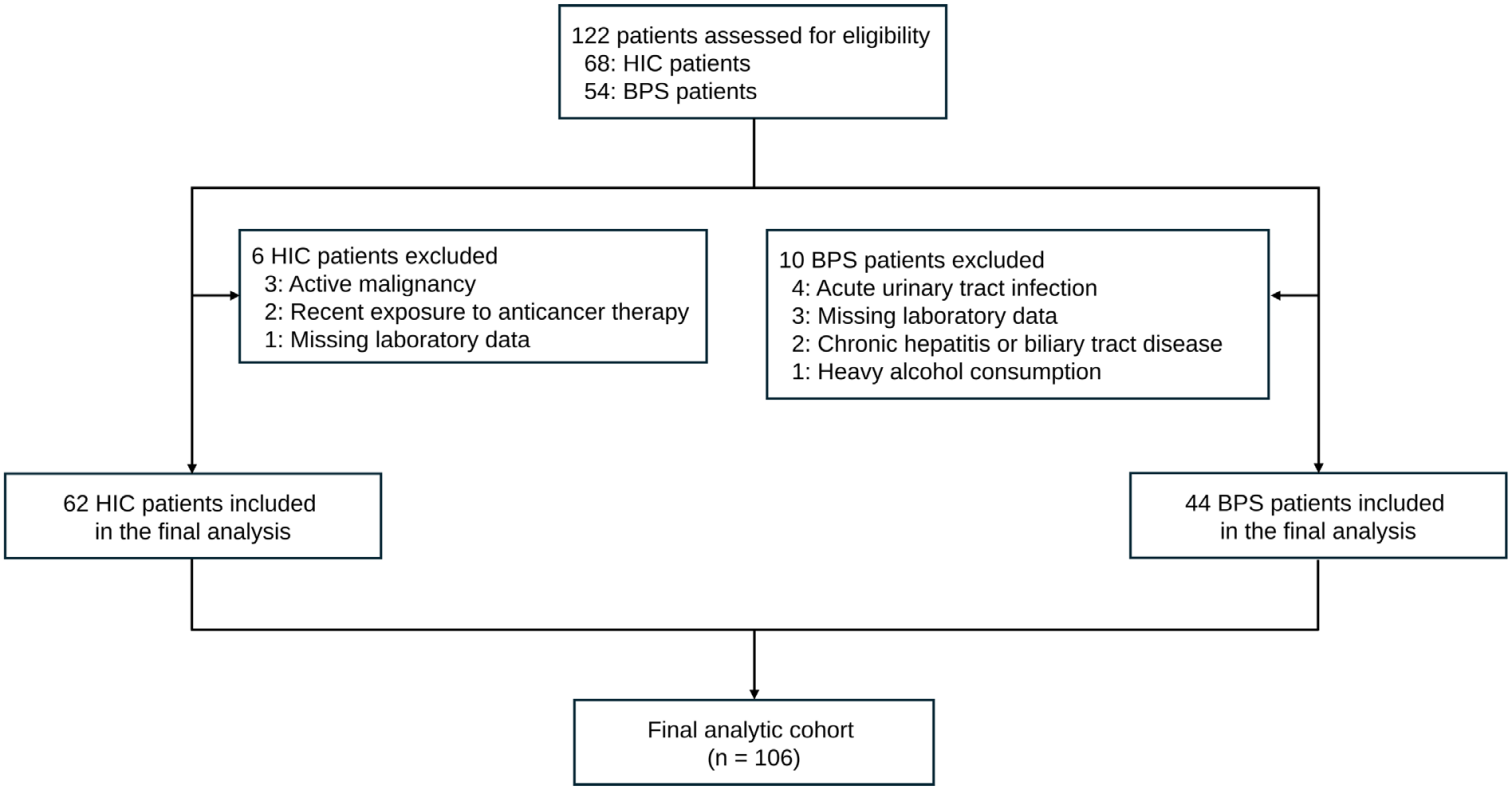
Patient flow diagram. A total of 122 consecutive patients who underwent cystoscopic evaluation with hydrodistension for suspected IC/BPS were assessed for eligibility. After exclusion according to prespecified criteria, 106 patients were included in the final analysis, comprising 44 patients with BPS and 62 patients with HIC. BPS, bladder pain syndrome; HIC, Hunner‐type interstitial cystitis; IC/BPS, interstitial cystitis/bladder pain syndrome.

Consecutive patients who underwent cystoscopic evaluation with hydrodistension for suspected IC/BPS at Nagasaki University Hospital between 1 April 2016 and 30 September 2025 were screened. Of 122 assessed patients, 106 were included in the final analysis after applying the prespecified exclusion criteria (Figure 1). Patients were excluded for acute urinary tract infection, chronic hepatitis or biliary tract disease, heavy alcohol consumption, active malignancy, recent anticancer therapy, or missing laboratory data required for GLR calculation. Heavy alcohol consumption was defined according to Health Japan 21 (the Third Term) as an average daily pure alcohol intake of at least 40 g for men or at least 20 g for women [13].

### 2.2 Cystoscopic Evaluation and Diagnostic Definitions

All patients underwent cystoscopy with hydrodistension under general or spinal anesthesia. Bladder biopsies were obtained before hydrodistension from suspected Hunner lesions or, in patients without suspected lesions, from the posterior bladder wall. Hydrodistension was performed with normal saline at 80 cm H_2_O, capped at 800 mL, and maintained for 2 min per cycle for a total of 3 cycles.

Final diagnosis was determined by expert adjudication based on cystoscopic findings, clinical presentation, and bladder biopsy results. Two experienced investigators (T.M. and K.A.) independently reviewed all available information while blinded to GLR and other laboratory values, and disagreements were resolved by consensus. HIC was diagnosed when Hunner lesions were identified on cystoscopy and the clinicopathological assessment was consistent with IC in the narrow sense. BPS was diagnosed when bladder pain symptoms were present without Hunner lesions, biopsy findings did not support HIC, and other confusable diseases were excluded.

### 2.3 Histopathological Semi‐Quantitative Assessment

**FIGURE 2:**
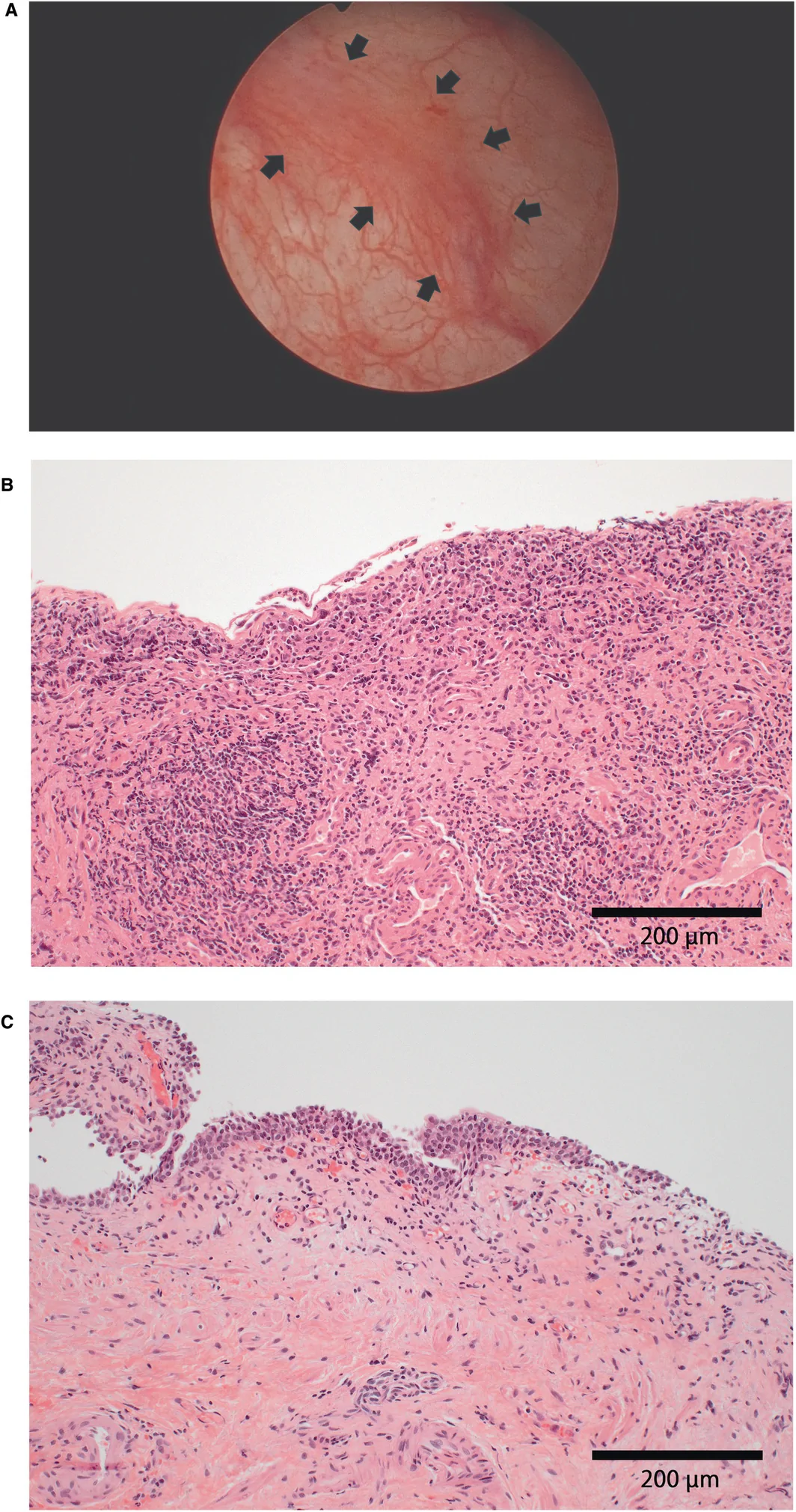
Representative cystoscopic and histopathological findings. (A) Representative cystoscopic image of a Hunner lesion in a patient with HIC. The area delineated by arrows indicates the Hunner lesion. (B) Hematoxylin and eosin‐stained bladder biopsy specimen from a patient with HIC showing urothelial denudation, chronic inflammatory cell infiltration, and stromal fibrosis. (C) Hematoxylin and eosin‐stained bladder biopsy specimen from a patient with BPS showing relatively preserved urothelium and less prominent inflammatory changes. Scale bars in B and C = 200 μm. HIC, Hunner‐type interstitial cystitis; BPS, bladder pain syndrome.

Based on previously reported histopathological features of IC/BPS and Hunner‐type IC [14, 15] and semi‐quantitative assessment methods for bladder histopathology [16], urothelial denudation, chronic inflammatory cell infiltration, and stromal fibrosis were evaluated using a semi‐quantitative 0–3 scale with minor modifications. These three components were selected to reflect epithelial injury, local inflammatory activity, and stromal remodeling, respectively. Urothelial denudation was graded according to the proportion of the biopsy surface lacking intact urothelial lining: 0, none or minimal; 1, < 25%; 2, 25%–50%; and 3, > 50% or extensive denudation. Chronic inflammatory cell infiltration in the lamina propria was graded as follows: 0, absent or minimal; 1, mild focal infiltration; 2, moderate multifocal infiltration; and 3, dense band‐like or diffuse infiltration. Stromal fibrosis was graded according to the extent and density of collagenous stromal remodeling: 0, absent or within normal limits; 1, mild focal fibrosis; 2, moderate multifocal fibrosis; and 3, marked diffuse fibrosis with architectural distortion. A total histological activity score was calculated as the sum of these three components, ranging from 0 to 9, and was interpreted as a composite histopathological severity measure. For semi‐quantitative scoring, all hematoxylin and eosin‐stained bladder biopsy slides were independently reviewed by two experienced urologists (S.K. and T.M.) with expertise in IC/BPS and cystoscopic–pathological assessment. The two evaluators were blinded to GLR values and other laboratory data. Discrepancies in semi‐quantitative scores were resolved by consensus. Representative histopathological images used for Figure 2 and the corresponding figure legend were reviewed by a pathologist (H.H.) to confirm that urothelial denudation, chronic inflammatory cell infiltration, and stromal fibrosis were appropriately represented.

### 2.4 Laboratory and Clinical Assessments

Pre‐procedural blood tests were obtained 2–4 weeks before cystoscopy/hydrodistension as part of routine care. Serum GGT, peripheral lymphocyte count, and C‐reactive protein (CRP) were measured using standardized automated analyzers. Lymphocyte counts were expressed as ×10^9^/L. GLR was calculated by dividing serum GGT (U/L) by peripheral lymphocyte count (×10^9^/L).

Baseline symptoms were assessed using the Interstitial Cystitis Symptom Index (ICSI), Interstitial Cystitis Problem Index (ICPI), and numerical rating scale for pain. A 3‐day bladder diary was used to calculate mean voided volume, as a surrogate of functional bladder capacity and mean daily voiding frequency.

### 2.5 Statistical Analysis

Primary analyzes were performed using JMP 18 (SAS Institute Inc., Cary, NC, USA), with additional revision analyzes using Python 3.13. Continuous and categorical variables were compared using appropriate parametric/non‐parametric and chi‐square/Fisher tests. Diagnostic performance of GLR was evaluated by ROC analysis, with the Youden‐index cut‐off reported. Logistic regression assessed associations with HIC; the multivariable model included age, sex, hypertension, chronic kidney disease, CRP, and GLR. Spearman coefficients assessed correlations between GLR and histopathological scores, symptom scores, and mean voided volume. Additional ROC analyzes compared GLR with GGT, lymphocyte count, NLR, PLR, and LMR using DeLong tests. Incremental diagnostic information was assessed by comparing a clinical logistic model including mean voided volume, bladder pain, ICSI, and ICPI with and without GLR, using AUC comparison and likelihood‐ratio testing. Variance inflation factors assessed multicollinearity; because ICSI and ICPI were strongly correlated, models adding GLR to each clinical variable separately were also examined. Two‐sided *p* < 0.05 was considered significant.

## 3 Results

### 3.1 Patient Characteristics

A total of 106 patients were included, comprising 44 with BPS and 62 with HIC (Figure 1). Patients with HIC were older than those with BPS (64.6 ± 13.6 vs. 58.0 ± 18.9 years, *p* = 0.040). Sex distribution and body mass index did not differ between groups. Chronic kidney disease was more prevalent in the HIC group (40.3% vs. 20.5%, *p* = 0.036), whereas hypertension and dyslipidemia did not reach statistical significance (Table 1).

**TABLE 1:** Patient characteristics according to IC/BPS phenotype.

	Overall	BPS	HIC	*p*
Number of patients	106	44	62	
Age, years	61.9 ± 16.2	58.0 ± 18.9	64.6 ± 13.6	0.040
Sex (male/female)	28/78	11/33	17/45	0.781
BMI, kg/m^2^	22.4 ± 3.8	22.3 ± 3.7	22.5 ± 3.8	0.918
Comorbidity				
Hypertension (%)	32 (30.2)	9 (20.5)	23 (37.1)	0.066
Diabetes mellitus (%)	10 (9.4)	4 (9.0)	6 (9.7)	0.919
CKD (%)	34 (32.1)	9 (20.5)	25 (40.3)	0.036
Dyslipidemia (%)	23 (21.7)	6 (13.6)	17 (27.4)	0.090
Autoimmune disease (%)	16 (15.1)	5 (11.4)	11 (17.7)	0.366
Laboratory examination				
AST (U/L)	22.1 ± 7.8	20.9 ± 5.5	23.0 ± 9.1	0.550
ALT (U/L)	18.3 ± 8.8	17.1 ± 7.6	19.2 ± 9.5	0.174
ALP (U/L)	170.6 ± 97.0	179.4 ± 92.2	164.4 ± 100.5	0.356
CRP (mg/dL)	0.19 ± 0.70	0.14 ± 0.34	0.22 ± 0.87	0.156
GGT (U/L)	25.8 ± 21.7	19.9 ± 19.1	30.0 ± 22.6	0.017
Neutrophil count, ×10^9^/L	3.4 ± 1.6	3.3 ± 1.6	3.5 ± 1.6	0.380
Monocyte count, ×10^9^/L	0.35 ± 0.15	0.34 ± 0.13	0.36 ± 0.16	0.980
Platelet count, ×10^9^/L	225.4 ± 72.5	225.8 ± 71.3	225.1 ± 73.9	0.644
Lymphocyte count, ×10^9^/L	1.55 ± 0.56	1.62 ± 0.52	1.51 ± 0.59	0.182
GLR	18.4 ± 15.3	14.2 ± 15.2	21.4 ± 14.7	0.016
NLR	2.49 ± 1.58	2.20 ± 1.24	2.69 ± 1.76	0.175
PLR	163.4 ± 81.1	154.1 ± 68.5	170.1 ± 88.9	0.633
LMR	4.95 ± 2.08	5.29 ± 2.27	4.70 ± 1.92	0.258

Abbreviations: ALP, alkaline phosphatase; ALT, alanine aminotransferase; AST, aspartate aminotransferase; BMI, body mass index; BPS, bladder pain syndrome; CKD, chronic kidney disease; CRP, C‐reactive protein; GGT, gamma‐glutamyltransferase; GLR, gamma‐glutamyltransferase‐to‐lymphocyte ratio; HIC, Hunner‐type interstitial cystitis; LMR, lymphocyte‐to‐monocyte ratio; NLR, neutrophil‐to‐lymphocyte ratio; PLR, platelet‐to‐lymphocyte ratio.

Serum GGT was higher in the HIC group than in the BPS group (30.0 ± 22.6 vs. 19.9 ± 19.1 U/L, *p* = 0.017), whereas lymphocyte counts did not differ significantly (1.51 ± 0.59 vs. 1.62 ± 0.52 × 10^9^/L, *p* = 0.182). Consequently, GLR was higher in the HIC group (21.4 ± 14.7 vs. 14.2 ± 15.2, *p* = 0.016). No significant differences were observed for aspartate aminotransferase (AST), alanine aminotransferase (ALT), alkaline phosphatase (ALP), CRP, neutrophil‐to‐lymphocyte ratio (NLR), platelet‐to‐lymphocyte ratio (PLR), or lymphocyte‐to‐monocyte ratio (LMR) (Table 1).

### 3.2 Urological Parameters and Symptom Scores

Compared with the BPS group, the HIC group had a lower mean voided volume (114.6 ± 71.1 vs. 272.0 ± 86.2 mL, *p* < 0.001) and higher bladder pain scores (7.2 ± 2.1 vs. 3.5 ± 1.8, *p* < 0.001). Mean daily voiding frequency did not differ significantly between the HIC and BPS groups (14.2 ± 5.4 vs. 15.7 ± 4.7, *p* = 0.141). All ICSI and ICPI item scores and total scores were higher in the HIC group (both total scores *p* < 0.001) (Table 2).

**TABLE 2:** Clinical, urological, and histopathological parameters according to IC/BPS phenotype.

	Overall	BPS	HIC	*p*
Mean daily voiding frequency	14.8 ± 5.1	15.7 ± 4.7	14.2 ± 5.4	0.141
Mean voided volume (mL)	180.0 ± 109.8	272.0 ± 86.2	114.6 ± 71.1	< 0.001
Bladder pain, NRS 0–10	5.7 ± 2.7	3.5 ± 1.8	7.2 ± 2.1	< 0.001
ICSI‐1	2.5 ± 1.4	1.5 ± 0.9	3.3 ± 1.3	< 0.001
ICSI‐2	3.0 ± 1.6	1.6 ± 1.0	4.0 ± 1.0	< 0.001
ICSI‐3	3.0 ± 1.5	1.8 ± 1.0	3.9 ± 1.1	< 0.001
ICSI‐4	2.7 ± 1.5	1.6 ± 1.1	3.5 ± 1.2	< 0.001
ICSI total	11.3 ± 5.3	6.6 ± 3.1	14.6 ± 3.7	< 0.001
ICPI‐1	2.4 ± 1.1	1.6 ± 0.8	3.0 ± 0.9	< 0.001
ICPI‐2	2.4 ± 1.2	1.5 ± 0.8	3.0 ± 1.0	< 0.001
ICPI‐3	2.2 ± 1.3	1.3 ± 0.8	2.8 ± 1.1	< 0.001
ICPI‐4	2.3 ± 1.2	1.4 ± 0.9	3.0 ± 0.9	< 0.001
ICPI total	9.3 ± 4.2	5.8 ± 2.6	11.8 ± 3.2	< 0.001
Histopathological scores				
Urothelial denudation score	1.7 ± 1.1	0.6 ± 0.8	2.4 ± 0.6	< 0.001
Chronic inflammation score	1.9 ± 1.0	1.1 ± 0.9	2.5 ± 0.6	< 0.001
Stromal fibrosis score	1.6 ± 0.8	1.1 ± 0.8	1.9 ± 0.6	< 0.001
Total histological activity score	5.2 ± 2.6	2.8 ± 2.1	6.9 ± 1.3	< 0.001

Abbreviations: BPS, bladder pain syndrome; HIC, Hunner‐type interstitial cystitis; ICPI, Interstitial Cystitis Problem Index; ICSI, Interstitial Cystitis Symptom Index; NRS, numerical rating scale.

### 3.3 Histopathological Scores and Their Associations With GLR

Histopathological scores for urothelial denudation, chronic inflammatory cell infiltration, stromal fibrosis, and total histological activity were significantly higher in the HIC group than in the BPS group (all *p* < 0.001; Table 2). In the overall cohort, GLR was positively correlated with urothelial denudation score (*ρ* = 0.555, *p* < 0.001), chronic inflammatory cell infiltration score (*ρ* = 0.556, *p* < 0.001), stromal fibrosis score (*ρ* = 0.466, *p* < 0.001), and total histological activity score (*ρ* = 0.606, *p* < 0.001; Table 3). In subgroup analyzes, GLR was positively correlated with total histological activity score in both the BPS group (*ρ* = 0.701, *p* < 0.001) and the HIC group (*ρ* = 0.294, *p* = 0.0203; Table 3).

**TABLE 3:** Correlations between GLR and histopathological scores.

	*ρ*	*p*
Overall		
Urothelial denudation score	0.555	< 0.001
Chronic inflammation score	0.556	< 0.001
Stromal fibrosis score	0.466	< 0.001
Total histological activity score	0.606	< 0.001
BPS		
Urothelial denudation score	0.442	0.0027
Chronic inflammation score	0.648	< 0.001
Stromal fibrosis score	0.536	< 0.001
Total histological activity score	0.701	< 0.001
HIC		
Urothelial denudation score	0.304	0.0162
Chronic inflammation score	0.150	0.2455
Stromal fibrosis score	0.140	0.2782
Total histological activity score	0.294	0.0203

*Note:* Correlations were assessed using Spearman's rank correlation coefficient.

Abbreviations: BPS, bladder pain syndrome; GLR, gamma‐glutamyltransferase‐to‐lymphocyte ratio; HIC, Hunner‐type interstitial cystitis.

### 3.4 Diagnostic Performance and GLR‐Based Stratification

ROC analysis demonstrated that GLR discriminated HIC from BPS with an AUC of 0.759 (*p* = 0.005). An exploratory cut‐off of 13.02 yielded sensitivity 0.823 and specificity 0.727. Baseline characteristics and clinical, urological, and histopathological parameters by GLR category are shown in Tables S1 and S2. The high‐GLR group included more HIC patients (*p* < 0.001), lower mean voided volume (129.9 ± 91.7 vs. 253.3 ± 92.0 mL, *p* < 0.001), and higher bladder pain, ICSI, and ICPI scores (all *p* < 0.001). GLR correlated positively with bladder pain (*ρ* = 0.338), ICSI (*ρ* = 0.431), and ICPI (*ρ* = 0.416), and negatively with mean voided volume (*ρ* = −0.488; all *p* < 0.001).

Comparative ROC analysis yielded AUCs of 0.776 for GGT, 0.576 for lymphocyte count, 0.578 for NLR, 0.527 for PLR, and 0.565 for LMR; GLR and GGT did not differ (DeLong *p* = 0.592; Table S3). The clinical model including mean voided volume, bladder pain, ICSI, and ICPI had an AUC of 0.948 versus 0.949 after adding GLR (DeLong *p* = 0.879; likelihood‐ratio *p* = 0.637). Because ICSI and ICPI showed substantial collinearity (*ρ* = 0.915; VIFs 7.10 and 7.26), parsimonious analyzes were also performed; GLR did not significantly improve any model (Table S4).

### 3.5 Association Between GLR and HIC

In univariable logistic regression, age (OR 1.03, 95% CI 1.01–1.05, *p* = 0.040), chronic kidney disease (OR 2.63, 95% CI 1.11–6.68, *p* = 0.028), and GLR (OR 1.05 per 1‐unit increase, 95% CI 1.01–1.11, *p* = 0.005) were associated with HIC (Table 4). In the multivariable model, GLR remained associated with HIC after adjustment for age, sex, hypertension, chronic kidney disease, and CRP (OR 1.04, 95% CI 1.01–1.10, *p* = 0.032), whereas the other variables were not statistically significant.

**TABLE 4:** Logistic regression analyzes for the association between GLR and HIC.

	Univariable analysis			Multivariable analysis		
	Odds ratio	95% CI	*p*	Odds ratio	95% CI	*p*
Age, per 1‐year increase	1.03	1.01–1.05	0.040	1.02	0.99–1.05	0.247
Sex, male	1.13	0.47–2.79	0.780	1.36	0.50–3.76	0.543
Hypertension	2.29	0.96–5.85	0.062	1.84	0.72–4.91	0.202
CKD	2.63	1.11–6.68	0.028	1.83	0.70–5.03	0.222
CRP, mg/dL	1.22	0.67–4.00	0.539	1.02	0.53–3.36	0.959
GLR, per 1‐unit increase	1.05	1.01–1.11	0.005	1.04	1.01–1.10	0.032

*Note:* HIC was the dependent variable. The multivariable model included age, sex, hypertension, CKD, CRP, and GLR. GLR was analyzed as a continuous variable per 1‐unit increase.

Abbreviations: CI, confidence interval; CKD, chronic kidney disease; CRP, C‐reactive protein; GLR, gamma‐glutamyltransferase‐to‐lymphocyte ratio; HIC, Hunner‐type interstitial cystitis; OR, odds ratio.

## 4 Discussion

This retrospective study showed that pre‐procedural GLR, a routinely available blood‐based index reflecting redox–immune balance, was higher in HIC than in BPS and showed moderate discriminatory performance, with an AUC of 0.759. An exploratory cut‐off value of 13.02 yielded a sensitivity of 0.823 and specificity of 0.727. Patients with high GLR also had a more severe bladder‐centric profile, including lower mean voided volume and higher pain and ICSI/ICPI scores. Furthermore, GLR remained independently associated with HIC after adjustment for clinically relevant covariates. These findings suggest that GLR may serve as a pragmatic adjunct for assessing bladder inflammatory activity and supporting early phenotyping in patients with IC/BPS symptoms, rather than as a standalone diagnostic test or Hunner lesion‐specific biomarker.

Phenotype‐oriented diagnosis is increasingly emphasized in IC/BPS guidance, particularly the distinction between HIC and non–Hunner lesion disease [1, 2, 3, 7, 17]. HIC is regarded as a bladder‐centric inflammatory subtype with demonstrable bladder pathology, whereas non–Hunner lesion IC/BPS may include more heterogeneous symptom mechanisms [4, 17]. In this cohort, HIC was associated with lower mean voided volume and higher pain and symptom scores, consistent with meta‐analytic evidence showing more severe bladder‐centric manifestations in patients with Hunner lesions [2]. Because confirmation of Hunner lesion status generally relies on cystoscopy, a simple blood‐based marker that supports early prioritization for cystoscopic evaluation could have clinical value.

Biological plausibility for GLR is supported by evidence implicating oxidative stress and immune dysregulation in IC/BPS [5, 6, 18, 19, 20]. Oxidative stress can amplify inflammation, impair urothelial integrity, and promote neural sensitization [6, 19]. HIC has also been described as a distinct inflammatory disorder characterized by pancystitis and frequent clonal B‐cell expansion [14]. GGT is involved in glutathione homeostasis and has been linked to oxidative stress and cardiometabolic risk [9, 10], whereas lymphocyte count reflects systemic immune‐inflammatory status [11, 12]. Therefore, GLR may reflect systemic correlates of bladder inflammatory activity rather than cystoscopic Hunner lesions themselves.

The additional histopathological analysis further strengthened the biological interpretation of GLR. GLR was positively correlated with semi‐quantitative scores for urothelial denudation, chronic inflammatory cell infiltration, stromal fibrosis, and total histological activity in the overall cohort. These findings suggest that GLR may reflect, at least in part, local bladder inflammatory activity, epithelial injury, and tissue remodeling. Importantly, GLR should not be interpreted as a biomarker specific to Hunner lesions. The stronger correlation between GLR and histological activity in the BPS subgroup than in the HIC subgroup suggests that GLR may primarily reflect inflammatory activity and tissue injury across the IC/BPS spectrum. The higher GLR observed in HIC may therefore partly reflect the greater inflammatory burden associated with the HIC phenotype, rather than Hunner lesions per se. In contrast, correlations within the HIC subgroup were modest, possibly because histological inflammation and epithelial injury were already advanced in most HIC patients, resulting in a restricted scoring range.

Most IC/BPS biomarker studies have focused on urine‐ or bladder‐derived markers, including inflammatory, urothelial, and oxidative stress markers [4, 5, 21]. Matched serum‐ and urine‐based approaches also suggest that systemic markers can reflect phenotype‐related biology [8]. Unlike many candidate biomarkers requiring specialized assays, GLR can be calculated from routine blood tests. However, GGT alone showed discrimination similar to GLR, and adding GLR did not materially improve discrimination beyond routine clinical variables. Thus, the present data do not establish superiority of GLR over GGT or incremental diagnostic classification value. Its potential value may instead lie in providing a low‐burden systemic correlate of bladder inflammatory activity, complementary to clinical assessment and cystoscopy.

Several limitations should be noted. First, the retrospective single‐center design may introduce selection bias and unmeasured confounding. Second, the cohort was limited to patients undergoing cystoscopy with hydrodistension and biopsy and may not represent the full IC/BPS spectrum. Third, the sample size was modest for multivariable modeling. Fourth, the ROC‐derived cut‐off of 13.02 was developed and evaluated in the same single‐center dataset and therefore requires external validation. Fifth, although major factors affecting GGT were excluded and CRP was included in the multivariable model, residual confounding by smoking, subclinical hepatic steatosis, medications, metabolic syndrome, or other inflammatory conditions remains possible. GGT alone showed diagnostic performance similar to GLR, so the added value of the ratio over GGT itself was not established. Sixth, subtle Hunner lesions, phenotype‐dependent biopsy sites, and sampling limitations may have affected classification or histopathological severity. Although assessments were performed by experienced investigators blinded to GLR, formal interobserver reproducibility statistics were not calculated; histopathological correlations should therefore be considered exploratory. Because the summed histopathological score included stromal fibrosis, it represents composite severity rather than pure activity. Seventh, the 2–4‐week interval between blood sampling and cystoscopy could allow interval laboratory changes. Finally, treatment response, lesion extent, disease duration, and previous treatment were not assessed. Incremental analyzes were exploratory, performed in the same modest cohort, and affected by collinearity among symptom measures. Future prospective multicenter studies should use prespecified thresholds and external validation, with reporting in accordance with STARD 2015 [22], and integrate GLR with clinical questionnaires, voiding diaries, urine biomarkers, imaging, cystoscopy, and pathology [4, 8, 23, 24, 25], and examine whether GLR predicts treatment response or reflects inflammatory, tissue‐remodeling, and oxidative stress signatures [6, 14, 19, 26, 27, 28].

In conclusion, pre‐procedural GLR may be a practical adjunctive biomarker reflecting bladder inflammatory activity across the IC/BPS spectrum, but it should not be regarded as Hunner lesion‐specific; external validation is required before routine clinical implementation.

## Author Contributions

**Hiroko Hayashi:** data curation, writing – review and editing. **Shota Yamada:** investigation, writing – review and editing. **Asato Otsubo:** investigation, writing – review and editing. **Shota Kakita:** conceptualization, investigation, writing – review and editing, writing – original draft. **Ryoichi Imamura:** writing – review and editing, supervision. **Kyohei Araki:** methodology, investigation, data curation, writing – review and editing. **Kojiro Ohba:** data curation, project administration, writing – review and editing. **Kensuke Mitsunari:** project administration, writing – review and editing. **Tomohiro Matsuo:** conceptualization, writing – review and editing, writing – original draft, methodology, investigation, formal analysis, data curation.

## Ethics Statement

The study protocol was approved by the Ethics Committee of Nagasaki University Hospital (approval No. 20122138) and was conducted in accordance with the Declaration of Helsinki.

## Consent

The requirement for written informed consent was waived because of the retrospective study design, and an opt‐out option was provided via the institutional website.

## Conflicts of Interest

The authors declare no conflicts of interest.

## Supporting information


**Table S1:** Baseline characteristics according to GLR category.
**Table S2:** Clinical, urological, and histopathological parameters according to GLR category.
**Table S3:** Diagnostic performance of GLR, GGT, lymphocyte count, and other blood‐based inflammatory indices for discriminating HIC from BPS.
**Table S4:** Incremental diagnostic value of GLR beyond routine clinical variables for discriminating HIC from BPS.

## Data Availability

The data that support the findings of this study are available on request from the corresponding author. The data are not publicly available due to privacy or ethical restrictions.
