# Supplementary material for: Pre‐Procedural Gamma‐Glutamyltransferase‐To‐Lymphocyte Ratio as an Adjunctive Biomarker for Differentiating Hunner‐Type Interstitial Cystitis From Bladder Pain Syndrome
Source: Int J Urol. 2026 Sep 14;33(9):e70638. doi: 10.1111/iju.70638 (PMC13574296; doi:10.1111/iju.70638)
Supplement: Supplementary file 1 [file IJU-33-0-s001.docx]

Table S1 Baseline characteristics according to GLR category

|  | Overall | Low GLR group  (<13.02) | High GLR group  (≥13.02) | P value |
| --- | --- | --- | --- | --- |
| Number of patients | 106 | 43 | 63 |  |
| BPS/HIC | 44/62 | 32/11 | 12/51 | <0.001 |
| Age, years | 61.9±16.2 | 58.9±20.2 | 63.9±12.7 | 0.123 |
| Sex (male/female) | 28/78 | 9/34 | 19/44 | 0.371 |
| BMI, kg/m^2^ | 22.4±3.8 | 21.8±3.1 | 22.8±4.1 | 0.477 |
| Comorbidity |  |  |  |  |
| Hypertension (%) | 32 (30.2) | 9 (20.9) | 23 (36.5) | 0.086 |
| Diabetes mellitus (%) | 10 (9.4) | 2 (4.7) | 8 (12.7) | 0.164 |
| CKD (%) | 34 (32.1) | 11 (25.6) | 23 (36.5) | 0.237 |
| Dyslipidemia (%) | 23 (21.7) | 12 (27.9) | 11 (17.5) | 0.200 |
| Autoimmune disease (%) | 16 (15.1) | 6 (14.0) | 10 (15.9) | 0.786 |
| Laboratory examination |  |  |  |  |
| AST (U/L) | 22.1±7.8 | 20.2±4.9 | 23.5±9.1 | 0.093 |
| ALT (U/L) | 18.3±8.8 | 15.5±6.1 | 20.3±9.8 | 0.006 |
| ALP (U/L) | 170.6±97.0 | 190.7±90.9 | 156.9±99.3 | 0.043 |
| CRP (mg/dL) | 0.19±0.70 | 0.15±0.34 | 0.22±0.86 | 0.368 |
| GGT (U/L) | 25.8±21.7 | 14.3±4.6 | 33.7±25.1 | <0.001 |
| Neutrophil count, ×10^9^/L | 3.4±1.6 | 3.5±1.4 | 3.4±1.7 | 0.320 |
| Monocyte count, ×10^9^/L | 0.35±0.15 | 0.35±0.12 | 0.35±0.17 | 0.652 |
| Platelet count, ×10^9^/L | 225.4±72.5 | 225.1±71.0 | 225.5±74.1 | 0.707 |
| Lymphocyte count, ×10^9^/L | 1.55±0.56 | 1.8±0.5 | 1.4±0.5 | <0.001 |
| GLR | 18.4±15.3 | 8.1±2.5 | 25.4±16.4 | <0.001 |
| NLR | 2.49±1.58 | 2.04±1.00 | 2.79±1.84 | 0.018 |
| PLR | 163.4±81.1 | 133.3±55.4 | 184.7±89.0 | 0.001 |
| LMR | 4.95±2.08 | 6.00±1.84 | 4.50±2.13 | 0.002 |
| BPS, bladder pain syndrome; HIC, Hunner-type interstitial cystitis; BMI, body mass index; CKD, chronic kidney disease; AST, aspartate aminotransferase; ALT, alanine aminotransferase; ALP, alkaline phosphatase; CRP, C-reactive protein; GGT, gamma-glutamyltransferase; GLR, gamma-glutamyltransferase-to-lymphocyte ratio; NLR, neutrophil-to-lymphocyte ratio; PLR, platelet-to-lymphocyte ratio; LMR, lymphocyte-to-monocyte ratio | | | | |

Table S2 Clinical, urological, and histopathological parameters according to GLR category

|  | Overall | Low GLR group  (<13.02) | High GLR group  (≥13.02) | P value |
| --- | --- | --- | --- | --- |
| Mean daily voiding frequency | 14.8±5.1 | 15.4±4.9 | 14.4±5.3 | 0.248 |
| Mean voided volume (mL) | 180.0±109.8 | 253.3±92.0 | 129.9±91.7 | <0.001 |
| Bladder pain, NRS 0–10 | 5.7±2.7 | 4.2±2.2 | 6.6±2.5 | <0.001 |
| ICSI-1 | 2.5±1.4 | 1.8±1.1 | 3.0±1.5 | <0.001 |
| ICSI-2 | 3.0±1.6 | 1.9±1.3 | 3.8±1.2 | <0.001 |
| ICSI-3 | 3.0±1.5 | 2.3±1.4 | 3.6±1.4 | <0.001 |
| ICSI-4 | 2.7±1.5 | 1.9±1.3 | 3.2±1.4 | <0.001 |
| ICSI total | 11.3±5.3 | 7.9±4.3 | 13.5±4.6 | <0.001 |
| ICPI-1 | 2.4±1.1 | 1.8±0.9 | 2.8±1.1 | <0.001 |
| ICPI-2 | 2.4±1.2 | 1.7±1.0 | 2.9±1.0 | <0.001 |
| ICPI-3 | 2.2±1.3 | 1.4±1.0 | 2.7±1.2 | <0.001 |
| ICPI-4 | 2.3±1.2 | 1.7±1.0 | 2.7±1.2 | <0.001 |
| ICPI total | 9.3±4.2 | 6.7±3.3 | 11.1±3.8 | <0.001 |
| Histopathological scores |  |  |  |  |
| Urothelial denudation score | 1.7±1.1 | 0.9±0.9 | 2.3±0.9 | <0.001 |
| Chronic inflammation score | 1.9±1.0 | 1.2±1.0 | 2.4±0.7 | <0.001 |
| Stromal fibrosis score | 1.6±0.8 | 1.1±0.8 | 1.9±0.6 | <0.001 |
| Total histological activity score | 5.2±2.6 | 3.2±2.3 | 6.6±1.8 | <0.001 |
| GLR, gamma-glutamyltransferase-to-lymphocyte ratio; NRS, numerical rating scale; ICSI, Interstitial Cystitis Symptom Index; ICPI, Interstitial Cystitis Problem Index | | | | |

Table S3. Diagnostic performance of GLR, GGT, lymphocyte count, and other blood-based inflammatory indices for discriminating HIC from BPS

| Marker | AUC (95% CI) | Optimal threshold for HIC | Sensitivity | Specificity | P vs GLR |
| --- | --- | --- | --- | --- | --- |
| GLR | 0.759 (0.656–0.862) | ≥13.02 | 0.823 | 0.727 | — |
| GGT | 0.776 (0.681–0.870) | ≥18.00 | 0.806 | 0.682 | 0.592 |
| Lymphocyte count | 0.576 (0.465–0.688) | ≤1.60 | 0.726 | 0.455 | <0.001 |
| NLR | 0.578 (0.467–0.688) | ≥1.92 | 0.645 | 0.523 | 0.007 |
| PLR | 0.527 (0.415–0.639) | ≥196.63 | 0.290 | 0.841 | <0.001 |
| LMR | 0.565 (0.454–0.675) | ≤5.33 | 0.645 | 0.523 | 0.003 |
| Cut-offs were determined using the Youden index; P values represent paired DeLong comparisons with GLR. HIC, Hunner-type interstitial cystitis; BPS, bladder pain syndrome; AUC, area under the receiver operating characteristic curve; CI, confidence interval; GLR, gamma-glutamyltransferase-to-lymphocyte ratio; GGT, gamma-glutamyltransferase; NLR, neutrophil-to-lymphocyte ratio; PLR, platelet-to-lymphocyte ratio; LMR, lymphocyte-to-monocyte ratio. | | | | | |

Table S4. Incremental diagnostic value of GLR beyond routine clinical variables for discriminating HIC from BPS

| Clinical model | AUC, clinical model  (95% CI) | AUC, clinical model + GLR (95% CI) | ΔAUC | P for ΔAUC | P for GLR addition |
| --- | --- | --- | --- | --- | --- |
| Primary model: mean voided volume + bladder pain NRS + ICSI total + ICPI total | 0.948 (0.899–0.998) | 0.949 (0.900–0.998) | 0.0002 | 0.879 | 0.637 |
| Sensitivity model: mean voided volume | 0.905 (0.838–0.971) | 0.909 (0.844–0.975) | 0.0048 | 0.247 | 0.577 |
| Sensitivity model: bladder pain NRS | 0.893 (0.828–0.959) | 0.905 (0.844–0.967) | 0.0121 | 0.126 | 0.175 |
| Sensitivity model: ICSI total | 0.937 (0.883–0.991) | 0.942 (0.892–0.993) | 0.0055 | 0.155 | 0.416 |
| Sensitivity model: ICPI total | 0.924 (0.867–0.982) | 0.931 (0.877–0.985) | 0.0068 | 0.072 | 0.209 |
| ΔAUC indicates the change in AUC after adding GLR. P for ΔAUC was calculated using the paired DeLong test, and P for GLR addition using the likelihood-ratio test. Because ICSI and ICPI were strongly correlated (Spearman ρ = 0.915), single-variable sensitivity models were also assessed. AUC, area under the receiver operating characteristic curve; CI, confidence interval; NRS, numerical rating scale; ICSI, Interstitial Cystitis Symptom Index; ICPI, Interstitial Cystitis Problem Index; GLR, gamma-glutamyltransferase-to-lymphocyte ratio. | | | | | |
